# Implementation of Delayed Cord Clamping for 3 Min During Term Cesarean Sections Does Not Influence Maternal Blood Loss

**DOI:** 10.3389/fped.2021.662538

**Published:** 2021-06-22

**Authors:** Stefanie Celen, Emily J. J. Horn-Oudshoorn, Ronny Knol, Eline C. van der Wilk, Irwin K. M. Reiss, Philip L. J. DeKoninck

**Affiliations:** ^1^Department of Pediatrics, University Hospital Gent, Ghent, Belgium; ^2^Division of Neonatology, Department of Pediatrics, Erasmus MC University Medical Center Rotterdam, Rotterdam, Netherlands; ^3^Department of Obstetrics and Gynecology, Erasmus MC University Medical Center Rotterdam, Rotterdam, Netherlands

**Keywords:** umbilical cord clamping, perinatal stabilization, cesarean section, maternal outcomes, maternal blood loss, postpartum hemorrhage, term pregnancies, neonatal outcomes

## Abstract

**Background:** To assess maternal safety outcomes after a local protocol adjustment to change the interval of cord clamping to 3 min after term cesarean section.

**Design, Setting, and Patients:** A retrospective cohort study in a tertiary referral hospital (Erasmus MC, Rotterdam). We included pregnant women who gave birth at term after cesarean section. A cohort (Nov 2016–Oct 2017) prior to the protocol implementation was compared to a cohort after its implementation (Nov 2017–Nov 2018). The study population covered 789 women (*n* = 376 pre-cohort; *n* = 413 post-cohort).

**Interventions:** Implementation of a local protocol changing the interval of cord clamping to 3 min in all term births.

**Main outcome measures:** Primary outcomes were the estimated maternal blood loss and the occurrence of postpartum hemorrhage (blood loss >1,000 ml). Secondary outcomes included both maternal as well as neonatal outcomes.

**Results:** Estimated maternal blood loss was not significantly different between the pre-cohort and post-cohort (400 mL [300–600] vs. 400 mL [300–600], *p* = 0.52). The incidence of postpartum hemorrhage (26 [6.9%] vs. 35 (8.5%), OR 1.24, 95% CI 0.73–2.11) and maternal blood transfusion (9 [2%] vs. 13 (3%), OR 1.33, 95% CI 0.56–3.14) were not different. Hemoglobin change was significantly higher in the post-cohort (−0.8 mmol/L [−1.3 to −0.5] vs. −0.9 mmol/L [−1.4 to −0.6], *p* = 0.01). In the post-cohort, neonatal hematocrit levels were higher (51 vs. 55%, *p* = 0.004) and need for phototherapy was increased (OR 1.95, 95% CI 0.99–3.84).

**Conclusion:** Implementation of delayed cord clamping for 3 min in term cesarean sections was not associated with increased maternal bleeding complications.

## Introduction

Severe postpartum hemorrhage (PPH) is one of the most important contributors to maternal mortality, particularly in low resource countries (1, 2). To reduce maternal blood loss, active management of the third stage of labor has been recommended by the World Health Organization (WHO) since 2007, although it was already performed since the 1960s (2–4). Active management involves three components: (i) prophylactic administration of uterotonic drugs (oxytocin), (ii) controlled cord traction to support placental delivery, and (iii) massage of uterine fundus after placental delivery (2, 5). However, a critical review of this guideline showed that the benefit of this approach was completely attributed to the administration of oxytocin (1, 5–7). On the other hand, to minimize potential neonatal exposure to oxytocin, immediate cord clamping was incorporated into routine clinical practice (3).

In term infants, the placenta holds up to one-third of the total blood volume and immediate cord clamping would thus withhold this from the neonatal circulation (8). In contrast, delayed cord clamping optimizes placental transfusion and thus results in a higher neonatal blood volume (1, 8, 9). Placental transfusion follows a stepwise curve and is completed in two phases: (i) up to 30% of the blood volume is transferred in the first minute after birth, and (ii) the remaining 70% is transferred in the subsequent 2 min (9). Hence performing delayed cord clamping for at least 3 min after birth should be considered in each healthy neonate (10, 11). Additionally, immediate cord clamping appears to have a negative effect on cardiovascular adaptations occurring at birth that are better supported when the infant is still connected to the placenta (12, 13). Although healthy term neonates usually experience an uneventful neonatal transition, delayed cord clamping has been shown to improve iron reserves far beyond the first months after birth, and decrease the likelihood of developing anemia (10, 13–17).

In elective cesarean sections the risk of maternal blood loss is estimated higher, due to the uterine incision and the lack of contractions. Therefore, clinicians have a tendency of clamping the cord shortly after delivery. On the other hand, a recent cohort study demonstrated that immediate cord clamping during a cesarean section was associated with increased neonatal iron deficiency anemia at 12 and 58 months (18). Hence, optimizing placental transfusion by delaying cord clamping until after 3 min would be of particular interest in this population. Based on the above-mentioned recommendations, we have recently implemented delayed cord clamping for 3 min as routine practice for all term births. The aim of this study was to audit maternal safety outcomes in cesarean deliveries after this protocol adjustment.

## Methods

This is a single-center retrospective cohort study performed at Erasmus MC, University Medical Center (Rotterdam, The Netherlands) comparing two cohorts, before and after a protocol adjustment in November 2017. For both cohorts the study population consisted of all consecutive cesarean sections performed at ≥37 weeks' gestation over a 1-year period. The control group (pre-cohort) consisted of cases between Nov 2016–Oct 2017 and the cohort after the protocol adjustment (post-cohort) consisted of cases between Nov 2017–Nov 2018. The research protocol was approved by the local medical ethical committee (METC 2019–0530) and informed consent was waived due to the retrospective study design.

**Figure 1:**
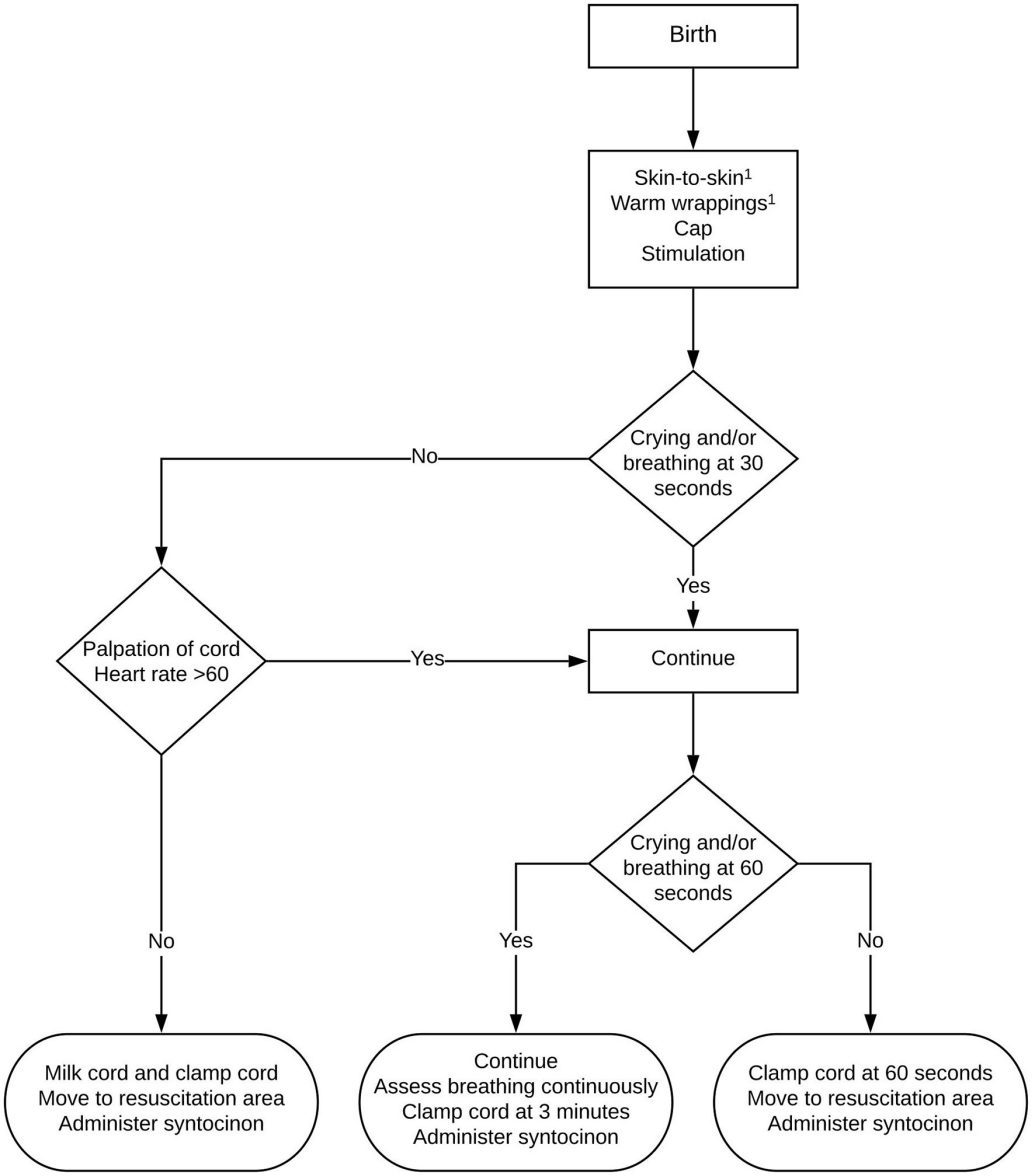
Flowchart of delayed cord clamping protocol at Erasmus MC, The Netherlands. ^1^During caesarean section: first dry, then stimulate. After cord clamping: skin-to-skin with warm wrappings or move to resuscitation area.

The protocol adjustment involved implementing delayed cord clamping for all term births for at least 3 min after complete birth of the infant. For all cesarean sections, the room temperature in the operation theater was increased to 23°C. After delivery, the infant was dried and positioned on the maternal chest close to the mother's face. Breathing was encouraged by tactile stimulation and the umbilical cord was protected from kinking or compression. The breathing rate and neonatal heart rate were evaluated after 30 s and 1 min by palpating the cord; when it was deemed appropriate and heart rate was approximately above 60 bpm the procedure was continued (Figure 1). The maternal blood loss was monitored continuously and, if necessary, hemostatic clamps were placed at the uterine incision. In case of excessive blood loss or concerns about the neonatal transition, the procedure was abandoned earlier and the infant was transferred to a resuscitation table for further support. If no exact time of cord clamping was mentioned in the post-cohort patient's history, we concluded that protocol had been followed and that delayed cord clamping of 3 min was performed. Oxytocin was administrated after umbilical cord clamping had been performed. The preferred method of placental delivery was spontaneous in both cohorts. All interventions were performed under direct supervision of a consultant in Obstetrics and Gynecology.

Predefined exclusion criteria for delayed cord clamping consisted of an emergency cesarean section (delivery warranted <10 min), maternal general anesthesia, monochorionic twin pregnancies, higher order multiple pregnancies, placental abnormalities (anterior placenta previa, invasive placentation), congenital diaphragmatic hernia, and severe cardiac anomalies. We excluded non-eligible cases from further analysis.

The primary outcomes were the estimated maternal blood loss and the occurrence of PPH (blood loss >1,000 ml) (19). Maternal blood loss was estimated by the obstetrician using the volume that was in the suction device and on the surgical swabs. Secondary outcomes included both maternal outcomes as well as neonatal outcomes. Maternal secondary outcomes consisted of hemoglobin change before and after cesarean section and the rate of blood transfusions. Neonatal secondary outcomes consisted of infant temperature at admission, moderate hypothermia (temperature <36°C), hematocrit level in the first 24 h after birth, neonatal intensive care unit admittance, and need for phototherapy in case of icterus neonatorum (confirmed by serum bilirubin sampling). Additionally, baseline characteristics of the neonate were evaluated such as birth weight, Apgar scores at 1 and 5 min, and umbilical artery pH. In both groups, an umbilical artery sample was obtained after the umbilical cord had been clamped.

Statistical analyses were performed using the computing environment R [R Core Team (2020), Vienna, Austria, v4.0.2]. Normality was assessed using QQ-plots and density distributions combined with the Shapiro-Wilk test. Continuous data are presented as mean ± standard deviation (SD) or median [interquartile range (IQR)], depending on whether the data were normally distributed. Categorical data are presented as absolute numbers and percentages. Statistical tests for continuous data were the Mann-Whitney U test (non-parametric) and the Student *t* test (parametric). Categorical variables were analyzed with a chi-squared test. Results are reported as odds ratio (OR) and 95% confidence interval (95% CI). We analyzed the effect of delayed cord clamping on maternal blood loss in the intention-to-treat and per protocol population. The intention-to-treat population is defined as all patients that were included in a particular group (pre-cohort vs. post-cohort), independent of protocol adherence. The per protocol population was defined as all patients who received the protocol they were assigned to (immediate cord clamping vs. delayed cord clamping at 3 min).

## Results

In the first cohort 659 cesarean sections were performed; 283 cases were excluded based on the predefined criteria, leaving 376 cases in the pre-cohort for further analysis. In the second cohort 699 cesarean sections were performed; 286 cases were excluded, leaving 413 cases in the post-cohort for further analysis. Table 1 presents the descriptive data of the mothers and neonates. Both groups were not different with regard to demographics and other clinical characteristics, except for umbilical artery blood gas analysis: pH (7.30 [7.26–7.32] vs. 7.28 [7.23–7.32], *p* < 0.001) and base excess (−2 mmol/L [−3 to 0] vs. −3 mmol/L [−5 to −1], *p* < 0.001) were significantly lower in the post-cohort.

**Table 1:** Baseline characteristics.

	***n***	**Pre-cohort**	***n***	**Post-cohort**
Maternal Age (y)	376	32 ± 5	413	32 ± 5
Nullipara	376	150 (40)	413	162 (39)
Elective cesarean section	376	235 (63)	413	264 (64)
Gestational age at delivery (wks)	376	39.0 [38.3–39.4]	413	39.0 [38.1–39.3]
Umbilical artery pH	368	7.30 [7.26–7.32]	399	7.28 [7.23–7.32]
Umbilical artery pCO_2_ (kPa)	363	7.20 [6.60–7.90]	399	7.20 [6.55–8.00]
Umbilical artery base excess (mmol/L)	362	−2 [−3 to 0]	395	−3 [−5 to −1]
Birthweight (g)	375	3327 ± 521	413	3349 ± 554
Apgar 1'	354	9 [8–9]	397	9 [8–9]
Apgar 5'	359	10 [9–10]	398	10 [9–10]

*Data are expressed as mean ± SD, median [IQR] or N (%)*.

In the post-cohort, the umbilical cord was clamped at 3 min in 53% (218/413) of the infants, at 30–170 s in 14% (59/413) of the infants, and immediately (<10 s) in 15% (63/413) of the infants. In 18% (73/413) of the infants, timing of cord clamping was not mentioned and we, thus, concluded that protocol was followed. Per protocol populations therefore consisted of 376 subjects for the immediate cord clamping group and 291 subjects for the delayed cord clamping group (3 min).

Table 2 shows the results of the primary outcomes in the intention-to-treat analysis. The median maternal blood loss did not significantly differ between pre-cohort and post-cohort (400 mL [300–600] vs. 400 mL [300–600], *p* = 0.52). In addition, there was no difference in the incidence of PPH: 26 mothers in the pre-cohort and 35 mothers in the post-cohort (OR 1.24, 95% CI 0.73–2.11). Per protocol analysis again demonstrated no difference in median maternal blood loss in immediate cord clamping compared to delayed cord clamping (400 mL [300–600] vs. 400 mL [300–500], *p* = 0.60) and no difference in the incidence of PPH (OR 0.78, 95% CI 0.41–1.48). Secondary maternal outcomes are presented in Table 3. Hemoglobin change was significantly different in the pre-cohort and post-cohort (−0.8 mmol/L [−1.3 to −0.5] vs. −0.9 mmol/L [−1.4 to −0.6], *p* = 0.01). The rate of maternal blood transfusions was not significantly different (OR 1.33, 95% CI 0.56–3.14).

**Table 2:** Primary outcomes.

	***n***	**Pre-cohort**	***n***	**Post-cohort**	
Maternal blood loss (mL)	375	400 [300–600]	413	400 [300–600]	*p* = 0.52
Maternal blood loss >1,000 mL	375	26 (6.9)	413	35 (8.5)	OR 1.24 (95% CI 0.73–2.11)

*Data are expressed as median [IQR] or N (%)*.

**Table 3:** Secondary outcomes.

	***n***	**Pre-cohort**	***n***	**Post-cohort**	
Maternal hemoglobin before delivery (mmol/L)	373	7.4 [6.9–7.9]	407	7.4 [6.9–7.9]	*p* = 0.90
Maternal hemoglobin after delivery (mmol/L)	366	6.4 [5.9–6.9]	408	6.4 [5.8–6.9]	*p* = 0.10
Maternal hemoglobin difference (mmol/L)	365	−0.8 [−1.3 to −0.5]	402	−0.9 [−1.4 to −0.6]	*p* = 0.01
Maternal blood transfusion	376	9 (2)	413	13 (3)	OR 1.33 (95% CI 0.56–3.14)
Neonatal intensive care unit admittance	376	79 (20)	413	79 (19)	OR 0.89 (95% CI 0.63–1.26)
Temperature of neonate (°C)	116	37.1 [36.7–37.3]	412	36.7 [36.3–37.0]	*p* <0.001
Hematocrit level of neonate (%)	69	51 ± 8	63	55 ± 8	*p* = 0.004
Phototherapy	376	13 (3)	413	27 (7)	OR 1.95 (95% CI 0.99–3.84)

*Data are expressed as mean ± SD, median [IQR] or N (%)*.

Neonatal outcomes are depicted in Table 3. Infant temperature at admission to the ward was lower in the post-cohort. The number of infants with moderate hypothermia was higher in the post-cohort [2/116 (2%) vs. 29/412 (7%), OR 4.32, 95% CI 1.01–18.36]. Neonatal hematocrit levels were significantly higher in the post-cohort, and the need for phototherapy was higher in the post-cohort (OR 1.95, 95% CI 0.99–3.84). The majority of infants receiving phototherapy had at least one serum bilirubin value that was equal to or higher than the intervention threshold for that specific infant: 85% (11/13) in the pre-cohort and 96% (26/27) in the post-cohort. The highest measured serum bilirubin values in infants receiving phototherapy were not different in both groups (286 μmol/L [230–323] vs. 297 μmol/L [263–320], *p* = 0.36).

There was no difference in rates of admission to the neonatal intensive care unit. In our tertiary care center population, 41% of all neonatal intensive care unit admissions were because of congenital malformations.

## Discussion

Our study adds information on the safety of delaying cord clamping until placental-to-neonatal transfusion is completed during term cesarean sections. In this study we did not observe differences in maternal blood loss and the incidence of PPH after implementing delayed cord clamping for 3 min as a routine practice. In addition, neonatal hematocrit levels in our trial were significantly higher after the implementation of delayed cord clamping, highlighting the importance of optimizing the placental-to-neonatal transfusion.

Our observations are in line with earlier trials that have already demonstrated that delayed cord clamping beyond 60 s during a vaginal delivery was not associated with increased maternal morbidity (4, 21). Nonetheless, the risk for maternal bleeding complications is estimated higher in cesarean section because of the lack of adequate uterine contractions. Hence, this resulted in a relative skepticism for implementing delayed cord clamping during a cesarean section. An important consideration is the delayed administration of oxytocin to avoid any influence on the placental-to-neonatal transfusion. However, a recent study showed that oxytocin could be administered prior to cord clamping without affecting the placental-to-neonatal transfusion (20). Previous studies evaluating delayed cord clamping in cesarean sections, though shorter durations (range 30–120 s), also did not report an increase in estimated maternal blood loss (21–24). In our study, the incidence of PPH remained stable after implementation of the protocol (6.9 vs. 8.5%) and reassuringly this is comparable to what is reported in a recent small randomized controlled trial comparing delayed cord clamping to immediate cord clamping in term cesarean delivery (23). Although we observed a significantly higher decrease in maternal hemoglobin after cesarean section, the clinical relevance of a difference of 0.1 mmol/L is certainly debatable. In particular as this difference did not translate into a higher incidence of maternal blood transfusions.

Neonates born through cesarean section are at higher risk for developing neonatal anemia since the placental-to-neonatal transfusion might be compromised because of a tendency to perform immediate cord clamping (1, 25). In our trial, we showed a benefit of delayed cord clamping on neonatal hematocrit levels, yet the optimal interval till clamping the cord is not firmly established. We based our protocol on a series of papers published more than 50 years ago, but others have observed a considerable individual variability in the time to complete placental transfusion (ranging between 2 and 5 min) (9, 26). Likewise, the impact of uterine contractions and breathing is not well-established (27). This is particularly important given that the potential side-effect of the additional blood volume is neonatal hyperbilirubinemia and the need for phototherapy, due to breakdown of red blood cells. The need for phototherapy in our study was higher in neonates after delayed cord clamping, as was also demonstrated in earlier trials (1, 8, 28).

Neonatal temperature management in the delivery room is of high importance, since hypothermia is associated with a higher risk of hypoglycemia and respiratory distress (29). Hypothetically, the umbilical blood is heated to the maternal core temperature after passage through the placenta, providing an internal heat source and, thus, potentially preventing hypothermia in delayed cord clamping. We could not confirm this hypothesis, on the contrary, we observed a significantly lower median infant temperature at admission to the ward after performing delayed cord clamping. In addition, we observed an increased incidence of moderate hypothermia, but the large number of missing data in the pre-cohort makes it difficult to draw firm conclusions, which is reflected by the large confidence interval. Nonetheless, our findings emphasize the importance of adequate measures to ensure optimal temperature management, especially during cesarean section. In our local protocol we have implemented additional measures such as sterile hats for the neonate and increased focus on drying the infant thoroughly.

We noticed a lower cord blood pH in the post-cohort. This was recently also reported in a prospective study evaluating the effect of delayed cord clamping on cord blood analysis in uncomplicated births. Delayed cord clamping at cesarean section resulted in a mixed respiratory and metabolic acidosis with increased pCO_2_ and lactate, in combination with a reduction in base excess (30–32). In our data, we confirmed the moderate decrease in base excess, but pCO_2_ values were not different. Regardless, this did not translate in increased adverse neonatal outcomes or neonatal intensive care unit admission rates. However, the incidence for the latter was in both groups remarkably higher than in previous trials (20 vs. 8%) (24). This is a reflection of the patient population at our institution as a tertiary referral center with a high incidence of congenital neonatal pathology.

The current study provides reassuring evidence about maternal safety when performing delayed cord clamping for a prolonged period in an attempt to improve placental-to-neonatal transfusion and thus neonatal iron reserves. This is important for future projects that aim to implement this in low resource settings, as the access to medications and equipment to control maternal bleeding is generally limited.

A strength of this study is that it evaluates a protocol implementation in a heterogenic group of patients that is a relevant representation of the general population for many centers. Furthermore, this study is the largest series so far evaluating the risks of delayed cord clamping during term cesarean sections and with a prolonged period of 3 min there was no higher risk of maternal bleeding complications. Limitations of adopting our findings into clinical practice are that this is a single-center trial in a high-income country in which we have easy access to drugs and equipment needed to control both maternal bleeding and the neonatal temperature. Estimating the amount of maternal blood loss was at the clinician's discretion, thereby introducing potential researcher bias. However, at the time of surgery, the clinicians were unaware of this study and, thus, the impact of this seems limited. Furthermore, we included objective variables evaluating the maternal blood loss such as maternal blood transfusions and hemoglobin differences. Protocol adherence in the post-cohort was not optimal with only 71% of the infants receiving delayed cord clamping for 3 min. Protocol adherence was similar in the first 6 months and the last 6 months of the post-cohort (69 vs. 72%). Reasons for early cord clamping were among others increased maternal bleeding and need for neonatal support. The retrospective design also introduces the risk of bias which is important to consider when interpreting the results, such as the incidence of moderate hypothermia. Around the same time of introducing delayed cord clamping, our hospital also altered its registration system for neonatal parameters. This new system resulted in improved documentation, explaining the difference with the cohort after protocol adjustment.

## Conclusions

Delayed cord clamping for 3 min during term cesarean sections appeared not to result in increased maternal bleeding complications. This study adds to the growing body of evidence assessing maternal safety when optimizing placental transfusion.

## Data Availability Statement

The raw data supporting the conclusions of this article will be made available by the authors, without undue reservation.

## Ethics Statement

The studies involving human participants were reviewed and approved by METC Erasmus MC. Written informed consent for participation was not provided by the participants' legal guardians/next of kin because: informed consent was waived due to the retrospective study design.

## Author Contributions

SC, EH-O, RK, EW, IR, and PD were all involved in the conception of this paper. SC and EH-O performed chart reviews and constructed the database for further analysis and wrote the first draft which was critically reviewed by all authors. SC, EH-O, RK, and PD contributed to the analysis and the interpretation of the results. All authors have approved the final version of the manuscript.

## Conflict of Interest

The authors declare that the research was conducted in the absence of any commercial or financial relationships that could be construed as a potential conflict of interest.

## References

[1] McDonald SJ Middleton P Dowswell T Morris PS. Effect of timing of umbilical cord clamping of term infants on maternal and neonatal outcomes. Cochrane Database Syst Rev. (2013) 2013:CD004074. 10.1002/14651858.CD004074.pub3 23843134 PMC6544813

[2] Winter C Macfarlane A Deneux-Tharaux C Zhang WH Alexander S Brocklehurst P. Variations in policies for management of the third stage of labour and the immediate management of postpartum haemorrhage in Europe. BJOG. (2007) 114:845–54. 10.1111/j.1471-0528.2007.01377.x 17567419 PMC1974828

[3] Begley CM Gyte GM Devane D McGuire W Weeks A Biesty LM. Active versus expectant management for women in the third stage of labour. Cochrane Database Syst Rev. (2019) 2:CD007412. 10.1002/14651858.CD007412.pub5 30754073 PMC6372362

[4] De Paco C Herrera J Garcia C Corbalan S Arteaga A Pertegal M. Effects of delayed cord clamping on the third stage of labour, maternal haematological parameters and acid-base status in fetuses at term. Eur J Obstet Gynecol Reprod Biol. (2016) 207:153–6. 10.1016/j.ejogrb.2016.10.031 27863273

[5] Rabe H Gyte GM Diaz-Rossello JL Duley L. Effect of timing of umbilical cord clamping and other strategies to influence placental transfusion at preterm birth on maternal and infant outcomes. Cochrane Database Syst Rev. (2019) 9:CD003248. 10.1002/14651858.CD003248.pub4 31529790 PMC6748404

[6] Salati JA Leathersich SJ Williams MJ Cuthbert A Tolosa JE. Prophylactic oxytocin for the third stage of labour to prevent postpartum haemorrhage. Cochrane Database Syst Rev. (2019) 4:CD001808. 10.1002/14651858.CD001808.pub3 31032882 PMC6487388

[7] Gallos ID Papadopoulou A Man R Athanasopoulos N Tobias A Price MJ. Uterotonic agents for preventing postpartum haemorrhage: a network meta-analysis. Cochrane Database Syst Rev. (2018) 12:CD011689. 10.1002/14651858.CD011689.pub3 30569545 PMC6388086

[8] Katheria AC Lakshminrusimha S Rabe H McAdams R Mercer JS. Placental transfusion: a review. J Perinatol. (2017) 37:105–11. 10.1038/jp.2016.151 27654493 PMC5290307

[9] Yao A Hirvensalo M Lind J. Placental transfusion-rate and uterine contraction. Lancet. (1968) 291:380–3. 10.1016/S0140-6736(68)91352-4 4169972

[10] Guideline: Delayed Umbilical Cord Clamping for Improved Maternal and Infant Health and Nutrition Outcomes. WHO Guidelines Approved by the Guidelines Review Committee. Geneva (2014). 26269880

[11] Wyllie J Bruinenberg J Roehr CC Rudiger M Trevisanuto D Urlesberger B. European resuscitation council guidelines for resuscitation 2015: section 7. resuscitation and support of transition of babies at birth. Resuscitation. (2015) 95:249–63. 10.1016/j.resuscitation.2015.07.029 26477415

[12] Hooper SB Polglase GR te Pas AB. A physiological approach to the timing of umbilical cord clamping at birth. Arch Dis Child Fetal Neonatal Ed. (2015) 100:F355–60. 10.1136/archdischild-2013-305703 25540147

[13] Qian Y Ying X Wang P Lu Z Hua Y. Early versus delayed umbilical cord clamping on maternal and neonatal outcomes. Arch Gynecol Obstet. (2019) 300:531–43. 10.1007/s00404-019-05215-8 31203386 PMC6694086

[14] Chaparro CM Neufeld LM Tena Alavez G Eguia-Liz Cedillo R Dewey KG. Effect of timing of umbilical cord clamping on iron status in Mexican infants: a randomised controlled trial. Lancet. (2006) 367:1997–2004. 10.1016/S0140-6736(06)68889-2 16782490

[15] Kc A Rana N Målqvist M Jarawka Ranneberg L Subedi K Andersson O. Effects of delayed umbilical cord clamping vs early clamping on anemia in infants at 8 and 12 months: a randomized clinical trial. JAMA Pediatr. (2017) 171:264–70. 10.1001/jamapediatrics.2016.3971 28114607

[16] Gyorkos TW Maheu-Giroux M Blouin B Creed-Kanashiro H Casapía M Aguilar E. A hospital policy change toward delayed cord clamping is effective in improving hemoglobin levels and anemia status of 8-month-old Peruvian infants. J Trop Pediatr. (2012) 58:435–40. 10.1093/tropej/fms012 22566383

[17] Andersson O Hellström-Westas L Domellöf M. Elective caesarean: does delay in cord clamping for 30 s ensure sufficient iron stores at 4 months of age? a historical cohort control study. BMJ Open. (2016) 6:e012995. 10.1136/bmjopen-2016-012995 27807089 PMC5129052

[18] Li HT Trasande L Zhu LP Ye RW Zhou YB Liu JM. Association of cesarean delivery with anemia in infants and children in 2 large longitudinal Chinese birth cohorts. Am J Clin Nutr. (2015) 101:523–9. 10.3945/ajcn.114.092585 25733637

[19] Anger H Durocher J Dabash R Winikoff B. How well do postpartum blood loss and common definitions of postpartum hemorrhage correlate with postpartum anemia and fall in hemoglobin? PLoS ONE. (2019) 14:e0221216. 10.1371/journal.pone.0221216 31437195 PMC6705817

[20] Vain NE Satragno DS Gordillo JE Fernandez AL Carrolli G Romero NP. Postpartum use of oxytocin and volume of placental transfusion: a randomised controlled trial. Arch Dis Child Fetal Neonatal Ed. (2020) 105:14–7. 10.1136/archdischild-2018-316649 31072967

[21] Andersson O Hellstrom-Westas L Andersson D Clausen J Domellof M. Effects of delayed compared with early umbilical cord clamping on maternal postpartum hemorrhage and cord blood gas sampling: a randomized trial. Acta Obstet Gynecol Scand. (2013) 92:567–74. 10.1111/j.1600-0412.2012.01530.x 22913332

[22] Kuo K Gokhale P Hackney DN Ruangkit C Bhola M March M. Maternal outcomes following the initiation of an institutional delayed cord clamping protocol: an observational case-control study. J Matern Fetal Neonatal Med. (2018) 31:197–201. 10.1080/14767058.2017.1280018 28068852

[23] Purisch SE Ananth CV Arditi B Mauney L Ajemian B Heiderich A. Effect of delayed vs immediate umbilical cord clamping on maternal blood loss in term cesarean delivery: a randomized clinical trial. JAMA. (2019) 322:1869–76. 10.1001/jama.2019.15995 31742629 PMC6865311

[24] Chantry CJ Blanton A Tache V Finta L Tancredi D. Delayed cord clamping during elective cesarean deliveries: results of a pilot safety trial. Matern Health Neonatol Perinatol. (2018) 4:16. 10.1186/s40748-018-0083-3 29997897 PMC6031144

[25] Cavallin F Galeazzo B Loretelli V Madella S Pizzolato M Visentin S. Delayed cord clamping versus early cord clamping in elective cesarean section: a randomized controlled trial. Neonatology. (2019) 116:252–9. 10.1159/000500325 31266035

[26] Farrar D Airey R Law GR Tuffnell D Cattle B Duley L. Measuring placental transfusion for term births: weighing babies with cord intact. BJOG. (2011) 118:70–5. 10.1111/j.1471-0528.2010.02781.x 21083868

[27] Boere I Roest AA Wallace E Ten Harkel AD Haak MC Morley CJ. Umbilical blood flow patterns directly after birth before delayed cord clamping. Arch Dis Child Fetal Neonatal Ed. (2015) 100:F121–5. 10.1136/archdischild-2014-307144 25389141

[28] Emhamed MO van Rheenen P Brabin BJ. The early effects of delayed cord clamping in term infants born to Libyan mothers. Trop Doct. (2004) 34:218–22. 10.1177/004947550403400410 15510946

[29] Laptook AR Watkinson M. Temperature management in the delivery room. Semin Fetal Neonatal Med. (2008) 13:383–91. 10.1016/j.siny.2008.04.003 18501693

[30] Giovannini N Crippa BL Denaro E Raffaeli G Cortesi V Consonni D. The effect of delayed umbilical cord clamping on cord blood gas analysis in vaginal and caesarean-delivered term newborns without fetal distress: a prospective observational study. BJOG. (2020) 127:405–13. 10.1111/1471-0528.16026 31762140

[31] Valero J Desantes D Perales-Puchalt A Rubio J Diago Almela VJ Perales A. Effect of delayed umbilical cord clamping on blood gas analysis. Eur J Obstet Gynecol Reprod Biol. (2012) 162:21–3. 10.1016/j.ejogrb.2012.01.020 22405491

[32] Wiberg N Källén K Olofsson P. Delayed umbilical cord clamping at birth has effects on arterial and venous blood gases and lactate concentrations. BJOG. (2008) 115:697–703. 10.1111/j.1471-0528.2008.01708.x 18410652

